# High gain low-cost antenna based on the utilization of diffracted fields from semi-ring shape dielectric edges

**DOI:** 10.1038/s41598-022-09288-5

**Published:** 2022-04-11

**Authors:** Yazan Al-Alem, Syed M. Sifat, Yahia M. M. Antar, Ahmed A. Kishk

**Affiliations:** 1 Electrical and Computer Engineering Department, Royal Military College of Canada, Kingston, ON Canada; 2 Electrical and Computer Engineering Department, Concordia University, Montreal, QC Canada; 3 Electrical and Computer Engineering Department, Queens University, Kingston, ON Canada

**Keywords:** Electrical and electronic engineering, Applied physics

## Abstract

A simple antenna with a 20-dBi gain is proposed. A thorough analysis of the propagation mechanism accompanied by a unique physical insight is provided. The realized structure has a low profile, low-cost, and compact features. A detailed insight into applying the Fresnel–Huygens principle is provided.

High gain antennas play a vital role in defining the performance limits of many wireless communication systems. High gain antennas are essential in compensating the path loss in wireless communication links^1–3^. There are many different types of structures and varieties of techniques that can provide high gain^4–7^. For example, reflector antennas are well known for their high directivity, and recently reflect/transmit-arrays as well^8–10^. Lenses can focus the radiation in the intended direction, and hence possess a high-gain performance^11^. These structures can achieve high performance metrics, but on the other hand, they are well-known for being non-planar and bulky, which make them not suitable for highly integrated systems. On the counterpart, planar antenna arrays are very well suited for integration and are low-profile. By doubling the number of elements in an array, an extra 3 dB can be obtained. As the size of the array (i.e., number of elements) increases, the size of the associated feeding network increases in a proportional manner. This increase in size not only increases the complexity of the design but also increases the number of discontinuities and junctions in the feeding structure. Consequently, the losses incorporated in such a feeding network increases^12–14^. Therefore, it is very valuable to find new techniques to minimize the size of the feeding network. In^15,16^, a unique design perspective to reduce the size of the feeding network was introduced, where a single slot antenna excitation provided a directivity of 16 dBi by utilizing diffracted fields from the edges of adjacent dielectric slabs. In this work we show that the dielectric slabs can take the shape of semi-rings to match the wave-front of an infinitesimal current source. In such case the radiation pattern can become a pencil-beam type of pattern rather than a fan-beam type pattern as in the case of rectangular dielectric slabs^15,17^. This has a great advantage where the directivity is improved significantly to go up to 22 dBi. Circular polarization operation is also thoroughly investigated. The proposed realization uses printed ridge gap waveguide technology, such technology is completely packaged, this improves the radiation characteristics by eliminating parasitic radiation from the feeding structure and suppresses any back-lobe radiation. Moreover, printed technology is more suitable for integrated systems, no bulky waveguides or transitions are needed, and hence it maintains a compact and low-profile type of solution.

## Huygens–Fresnel principle

**Figure 1:**
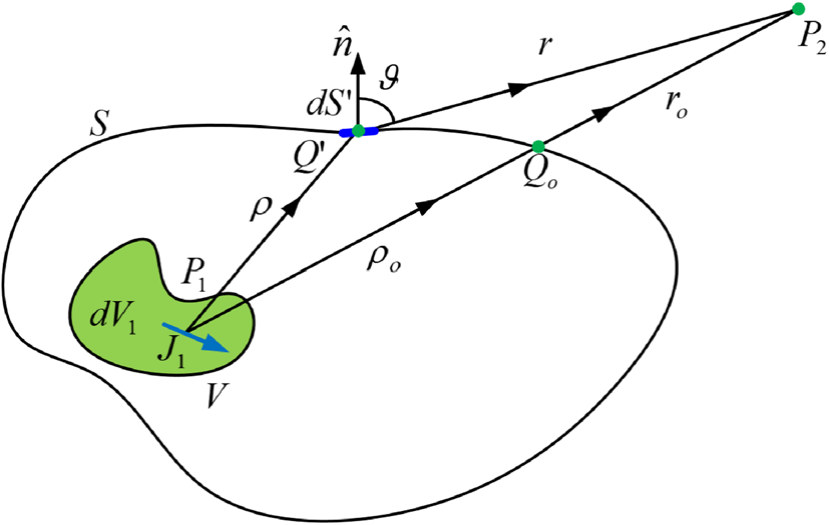
Huygens secondary wave surface enclosing the primary current source.

To dig deeper into the radiation mechanism of the structure, first we shall provide a brief introduction about Huygens-Fresnel principle. Huygens Principle is a powerful tool, where it gives an understanding of the mechanism of manipulating the radiation characteristics of any source. Figure 1 demonstrates the calculation of the fields of any arbitrary source using Huygens principle, the calculation of the fields can be obtained by the integral given by (2), where by knowing the field values on each point of an enclosing surface, the fields can be calculated directly at any arbitrary point outside the surface. The field values at each point on the arbitrary surface can be represented as a Huygens differential source as given by (1), the integration of these sources all over the surface using (2) allows the calculation of the fields outside the surface.

1 $$dE(P_{2} ) = E_{s} (Q^{\prime } )e^{{j\phi _{s} (Q^{\prime } )}} \frac{{e^{{j\beta r}} }}{r}I(\vartheta )dS^{\prime }$$

2 $$E(P_{2} ) = \iint\limits_{S} {E_{s} (Q^{\prime } )e^{{j\phi_{s} (Q^{\prime } )}} \frac{{e^{j\beta r} }}{r}I(\vartheta )dS^{\prime } }$$

**Figure 2:**
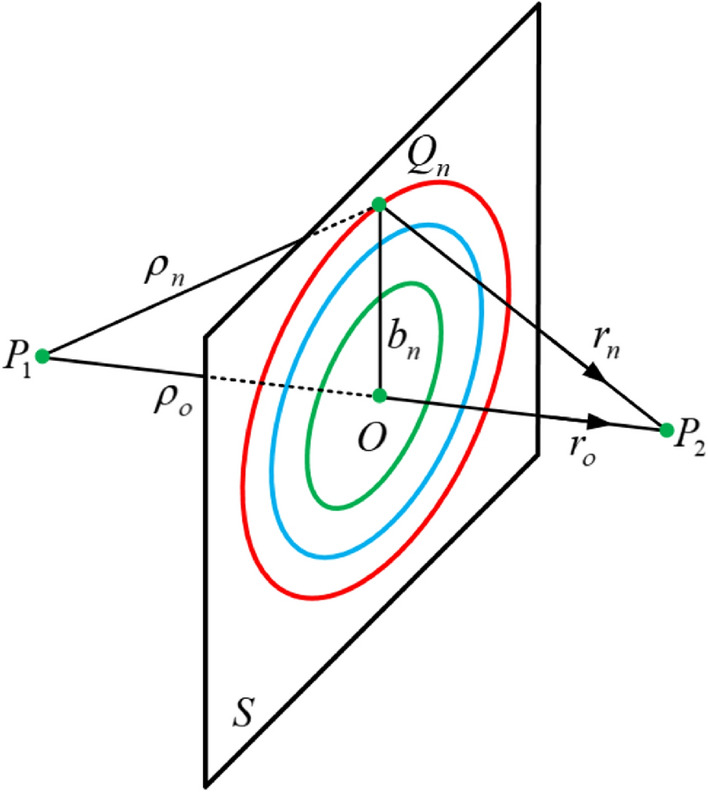
Fresnel zones calculation.

What makes Huygens method unique and powerful is that it provides us with a possibility to visualize how the fields can be manipulated by adding some absorbers/reflectors to eliminate some differential Huygens sources to achieve a certain desired field distribution. Consequently, the work of Huygens inspired Sorte to invent his famous Sorte focusing lens, the lens depends on calculating Fresnel zones, and then block out of phase zones to focus the radiation, the blockage can be performed by inserting a complete absorber or reflector in the desired zones. The calculation of Fresnel zones can be obtained as in Fig. 2, and Eqs. (3–10)^18–24^. In Fig. 2, it is assumed that a spherical source exists at *P*_1_, the plane *S* is an infinite plane surface, in such case there is an infinite concentric circles with radii *b*_*n*_ (i.e. phase contour lines) at which the phases of the Huygens sources add up constructively in phase with the direct line of sight radiation from *P*_1_ at the point *P*_2_. In such case the distance *r*_*o*_ is denoted as the focal point at which all the sources generated at the zone add up constructively.

3 $$\rho_{n} = \sqrt {b_{n}^{2} + \rho_{o}^{2} }$$

4 $$r_{n} = \sqrt {b_{n}^{2} + r_{o}^{2} }$$

5 $$\sqrt {b_{n}^{2} + \rho_{o}^{2} } + \sqrt {b_{n}^{2} + r_{o}^{2} } = \rho_{o} + r_{o} + \frac{n\lambda }{2}$$

6 $$\left( {\sqrt {b_{n}^{2} + \rho_{o}^{2} } + \sqrt {b_{n}^{2} + r_{o}^{2} } } \right)\left( {\sqrt {b_{n}^{2} + \rho_{o}^{2} } - \sqrt {b_{n}^{2} + r_{o}^{2} } } \right) = \rho_{o}^{2} - r_{o}^{2}$$

7 $$\begin{aligned} \left( {\sqrt {b_{n}^{2} + \rho_{o}^{2} } - \sqrt {b_{n}^{2} + r_{o}^{2} } } \right) & = \frac{{\rho_{o}^{2} - r_{o}^{2} }}{{\left( {\sqrt {b_{n}^{2} + \rho_{o}^{2} } + \sqrt {b_{n}^{2} + r_{o}^{2} } } \right)}} \\ & = \frac{{\rho_{o}^{2} - r_{o}^{2} }}{{\rho_{o} + r_{o} + \frac{n\lambda }{2}}} \\ \end{aligned}$$

By adding (5) and (7) we get (8) as follows:

8 $$2\sqrt {b_{n}^{2} + \rho_{o}^{2} } = \rho_{o} + r_{o} + \frac{n\lambda }{2} + \frac{{\rho_{o}^{2} - r_{o}^{2} }}{{\rho_{o} + r_{o} + \frac{n\lambda }{2}}}$$

9 $$b_{n} = \frac{1}{2}\sqrt {\left( {\rho_{o} + r_{o} + \frac{n\lambda }{2}} \right)^{2} + \left( {\frac{{\rho_{o}^{2} - r_{o}^{2} }}{{\rho_{o} + r_{o} + \frac{n\lambda }{2}}}} \right)^{2} - 2\left( {\rho_{o}^{2} + r_{o}^{2} } \right)}$$

If the distance *ρ*_*o*_ → ∞, and *r*_*o*_ = *F*, the incident wave impinging on the plane *S* becomes a plane wave and (9) can be reduced to (10).

10 $$b_{n} = \sqrt {n\lambda F + \left( {\frac{n\lambda }{2}} \right)^{2} }$$

**Figure 3:**
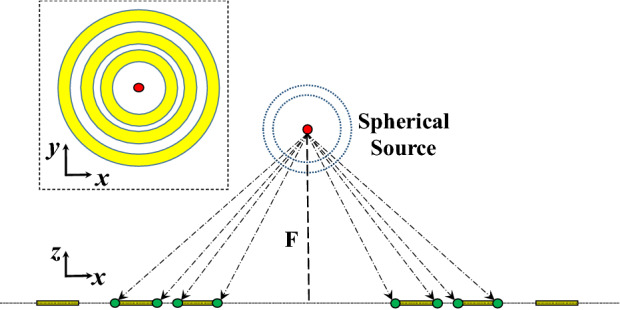
Fresnel zones and Spherical Source Excitation.

Practically the focal length should be electrically large. Figure 3 shows an illustration of a spherical source with respect to the Fresnel zones, the yellow region indicates the region of the absorbers/reflectors. The Focal length should be large to prevent the interaction of the absorbers/reflectors with the spherical source, “practically the spherical source is realized by a Horn, an Open-end waveguide, a Magneto-Electric (ME) dipole, etc.”. When the focal length is small, the close proximity of the source from the Fresnel zone (absorbers/reflectors) alters the input impedance of the source, and most importantly it distorts the spherical radiation behavior of the source. In such a case, the higher the interaction, the less spherical the source is, and this means the expected radiation characteristics and the boost in the gain cannot be achieved.

**Figure 4:**
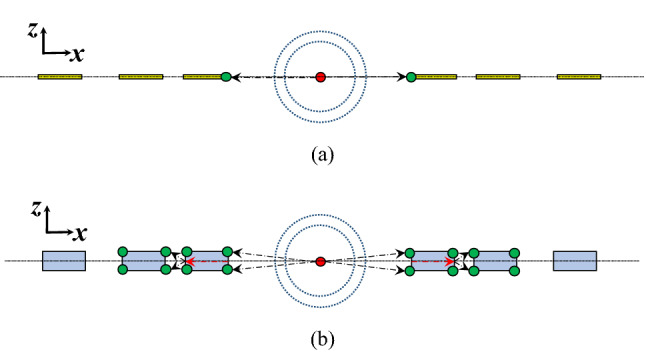
Fresnel zones and Spherical Source Excitation (F → 0), (**a**) Reflector/Absorber Case, and (**b**) Dielectric Case.

This is well understood by the fact that the Fresnel zones derivation is based on the assumption of an ideal spherical source. Within this context, it is worth emphasizing that the proposed structure is fully printed, low cost, and low profile as well. It does not require any bulky waveguides or transitions, also the method does not require a spherical feed such as a Horn or an ME dipole antenna. Ordinary slots and dipoles can be used. In addition, it is worth noting that in the case of a large focal length, the phase center of the antenna has to be determined to achieve a proper design. In the proposed case this requirement is not needed. In (11) we calculate the Fresnel zones when the focal length approaches zero. Figure 4 shows the spherical source for such scenario (i.e. when the focal length approaches zero), there are two cases, (a) when reflector/absorber sheets are used, and (b) when dielectric slabs are used instead of the reflector/absorber sheets. As can be seen, in case (a), the absorbers/reflectors prevent the spherical source wave from exciting the edges of the sheets beyond the first loop, and hence Huygens sources cannot be generated as expected. Also, it is worth noting that the spherical source polarization is ignored in the derivation of the Fresnel zones. In the case of absorbers the waves cannot propagate beyond the first loop as the first ring absorber will absorb the wave. The absorber scenario is not commonly used due to its realization complexity. For the Case of the reflector we end up with two scenarios, the first, if the source has a TE polarization “with respect to the *z*-axis in Fig. 4”, such case is equivalent to an electric dipole source, the reflector (PEC) sheets will act as soft surfaces and hence the waves will be suppressed by the first Fresnel loop.

The second scenario is when the source has a TM polarization, equivalently a slot antenna (i.e. magnetic current source), in this case the reflector sheets will act as a hard surface and allow the wave to propagate beyond the first loop, the tricky part is with the realization, the realization of a slot requires the slot to exist at the level of a metallic surface, also for a unidirectional radiation a PMC is needed to back the structure (usually realized by a corrugated surface or a periodic structure). In many cases a single grooved metal is sufficient to produce a high impedance equivalent to PMC, as the distance between the edges of grooves “Fresnel zone” is a fraction of a wavelength^25^. In case (b), the polarization of the source is not of a concern anymore, as the wave can penetrate the dielectric slabs and generate Huygens sources in the next zones regardless the polarization is TE or TM. This is a significant advantage that relaxes the realization significantly. As will be shown later in the manuscript this concept can be realized easily in printed technology without resorting to metallic milling.

11 $$R_{n} = \mathop {\lim }\limits_{F \to 0} \left( {\sqrt {n\lambda F + \left( {\frac{n\lambda }{2}} \right)^{2} } } \right) = \frac{n\lambda }{2}$$

## Infinitesimal current excitation between dielectric slabs

**Figure 5:**
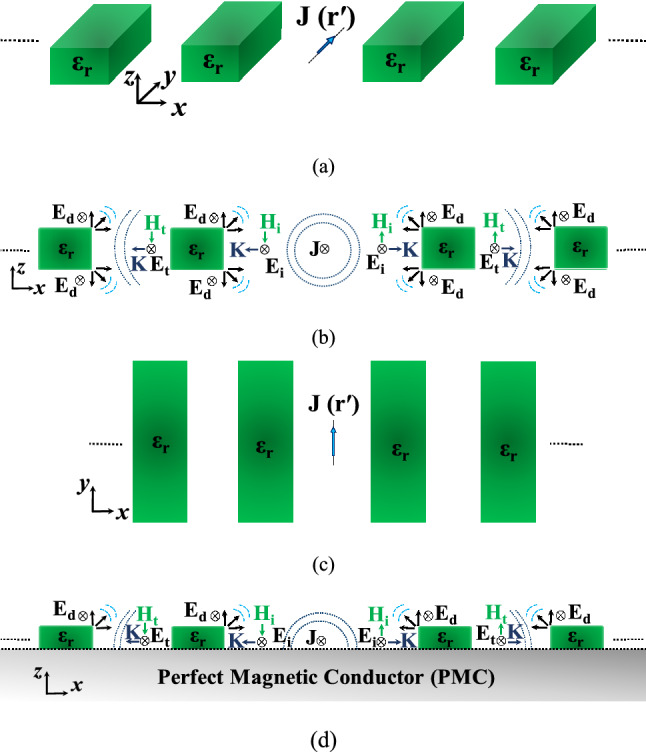
Electric current source in between dielectric slabs, (**a**) rectangular dielectric slabs “isometric view”, (**b**) side-view “propagation mechanism”, (**c**) top-view, and (**d**) unidirectional radiation “backed by PMC”.

Figure 5a shows an isometric view of an infinitesimal current source located in between several dielectric slabs. Figure 5b shows the side view in the *xz*-plane illustrating the propagation mechanism. Figure 5c shows the top view. As the current source radiates, the waves impinges on the first adjacent dielectric slabs, portion of the wave propagates through the dielectric slab and another portion diffracts from the edges of the slab. The diffracted fields from the edges radiate, hence by controlling the distance of the dielectric slab from the current source, the diffracted fields phases can be controlled to radiate in phase with the main current source, in such a way the directivity can be increased in the boresight direction. The other portion of the wave propagating through the dielectric slab can be transmitted fully to the other side of the slab, where it is re-radiated to illuminate the next slab, by controlling the size of the dielectric slab along the *x*-aixs to be multiple of a half guided wavelength, the transmission coefficient of the dielectric slab is maximized. As the next slab gets illuminated by its adjacent slab, diffracted fields from its edges are generated, and hence by controlling the distance from the illuminating slab, the phases of those diffracted fields are controlled to radiate in phase with the first slab diffracted fields and the main current source, and hence further boost the directivity in the boresight. As the number of slabs increases the energy of the transmitted waves through the slabs gradually decreases until it dies out. This has an advantage in tapering the power of the diffracted fields and hence it maintains an adequate side lobe level in the radiation pattern.

**Figure 6:**
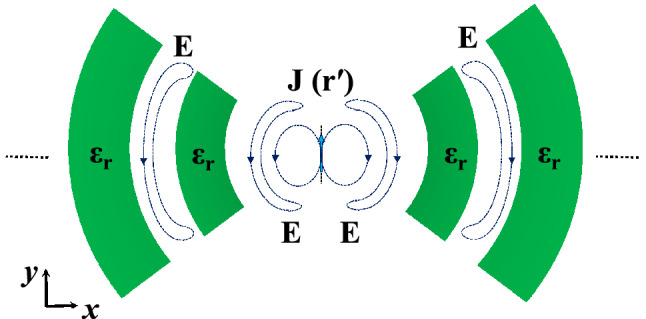
Electric current source in between dielectric slabs, semi-ring shape.

The configuration in Fig. 5a radiates along both the positive and negative sides of the *z*-axis, to make the radiation unidirectional towards the boresight, a Perfect Magnetic Conductor (PMC) can be used to truncate the geometry in half according to image theory as shown in Fig. 5d. The use of rectangular dielectric slabs can alter the radiation characteristics only along the *x*-axis, and hence the resultant radiation pattern is a fan-beam type of pattern. In Fig. 6 the dielectric slabs are bent in a semi-ring fashion to match the wave-front of the infinitesimal current source. As it will be shown later, this can alter the radiation characteristics in both planes to obtain a pencil-beam type pattern with further improved gain.

**Figure 7:**
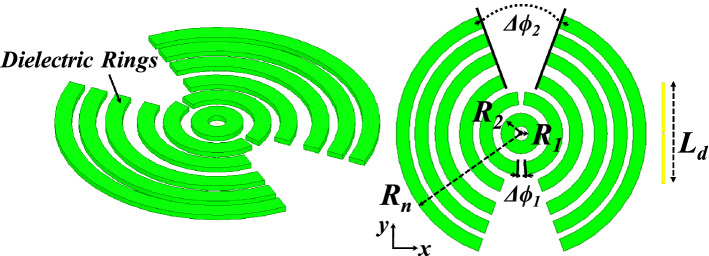
Electric current source in between dielectric slabs, semi-ring shape, right (top view), and left (isometric view).

**Figure 8:**
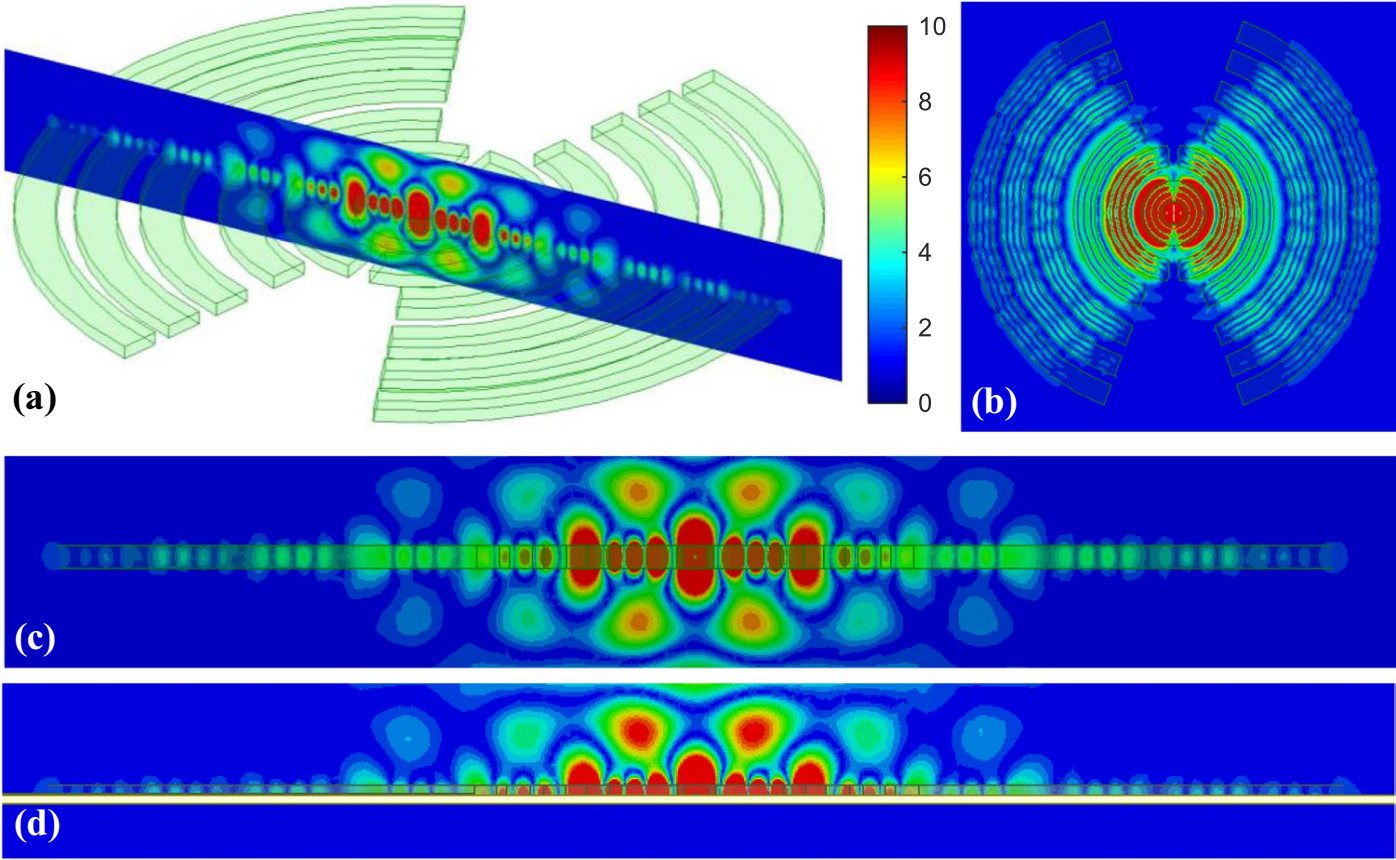
Electric field heat map of the proposed structure in Fig. 7, (**a**) isometric view, (**b**) top view, (**c**) h-plane cut, and (**d**) backed by PMC, scale (V/m).

**Figure 9:**
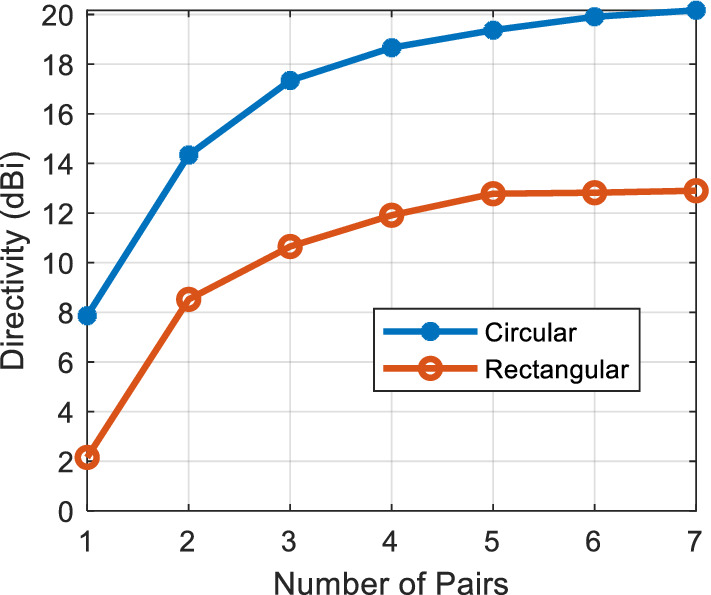
Directivity for each number of dielectric slabs pairs.

**Figure 10:**
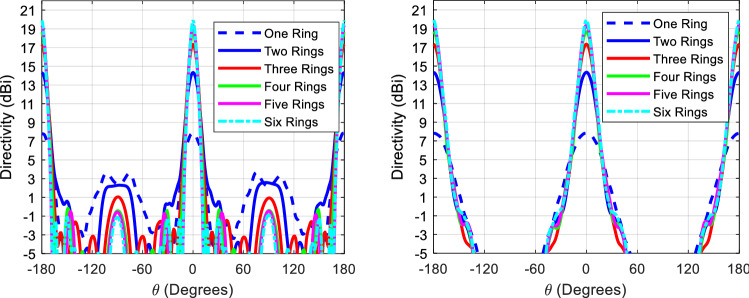
Radiation patterns for the proposed structure in Fig. 7 at 20 GHz, left (H-plane), and right (E-plane).

Figure 7 shows the 3D model used in HFSS to study the behavior of the proposed concept in Fig. 6. The dielectric rings are made of Rogers 6010 with a dielectric constant of 10.2 and a loss tangent of 0.0023. The height of each slab is H_d_. The complete list of dimensions are listed in Table 1. Figure 8 shows the electric field heat map of the proposed structure in Fig. 7. It is well-observed that the wave generated from the dipole is propagating through the dielectric slabs and diffracted from the edges, as well, the semi-ring shape matches well the wave-front generated from the dipole. Figure 8d shows the electric field heat map of the structure when it is truncated in half by a PMC surface in the *xy*-plane, as can be observed the intensity of the fields almost doubles as expected in the upper plane and gets suppressed in the back plane, hence obtaining a unidirectional radiation in the boresight direction. Figure 9 shows the directivity for each number of adjacent dielectric slabs, as can be seen the directivity increases gradually by the increase of the number of pairs until it saturates. Also as can be seen, there is almost a 6-dB difference in directivity between the rectangular shape and the semi-ring shape as expected. The semi-ring shape can carry more energy from the source to the last edge, as it utilizes the wave-front of the source unlike the rectangular shape. This would give the advantage of a higher gain and a lower saturation rate. Figure 10 shows the radiation patterns in principal planes, as can be noticed the radiation in the end-fire direction (i.e. 90°) gradually decreases by the increase of number of pairs, in addition to that the directivity increases as expected.

**Table 1:** Dimensions (millimeters).

R_1_	R_2_	R_3_	R_4_	R_5_	R_6_	R_7_	R_8_
5	14	20	29	34	43	51	60
R_9_	R_10_	R_11_	R_12_	Δϕ_1_	Δϕ_2_	L_d_	H_d_
65	74	78	87	5	20	6.7	3

**Figure 11:**
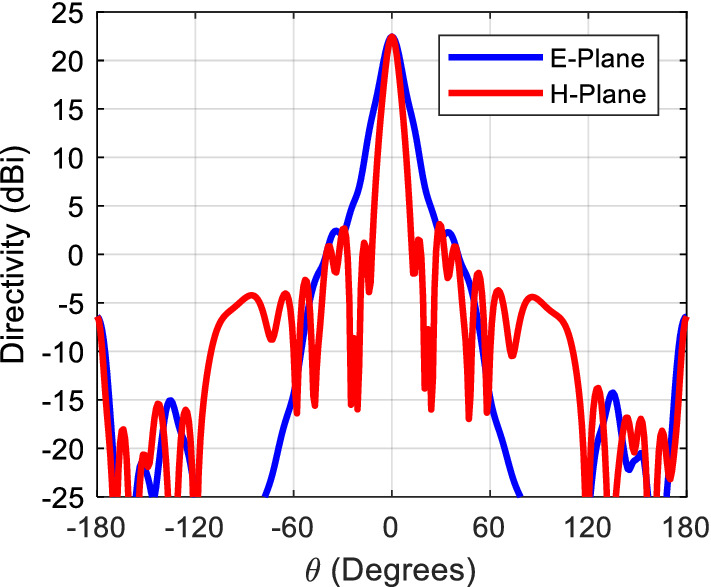
Radiation patterns for the proposed structure (PMC case) at 20 GHz.

**Figure 12:**
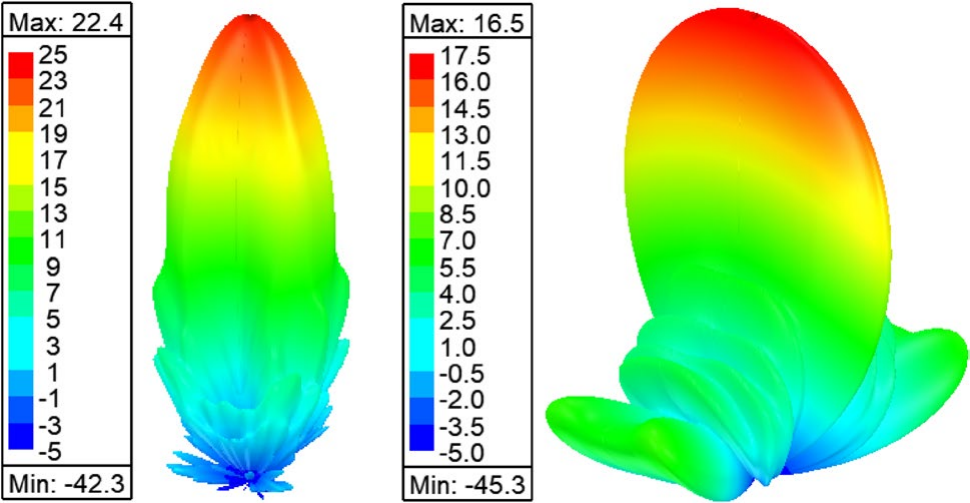
3D radiation pattern for the semi-ring shape dielectric slabs (left), and rectangular dielectric slabs (right) at 20 GHz.

**Figure 13:**
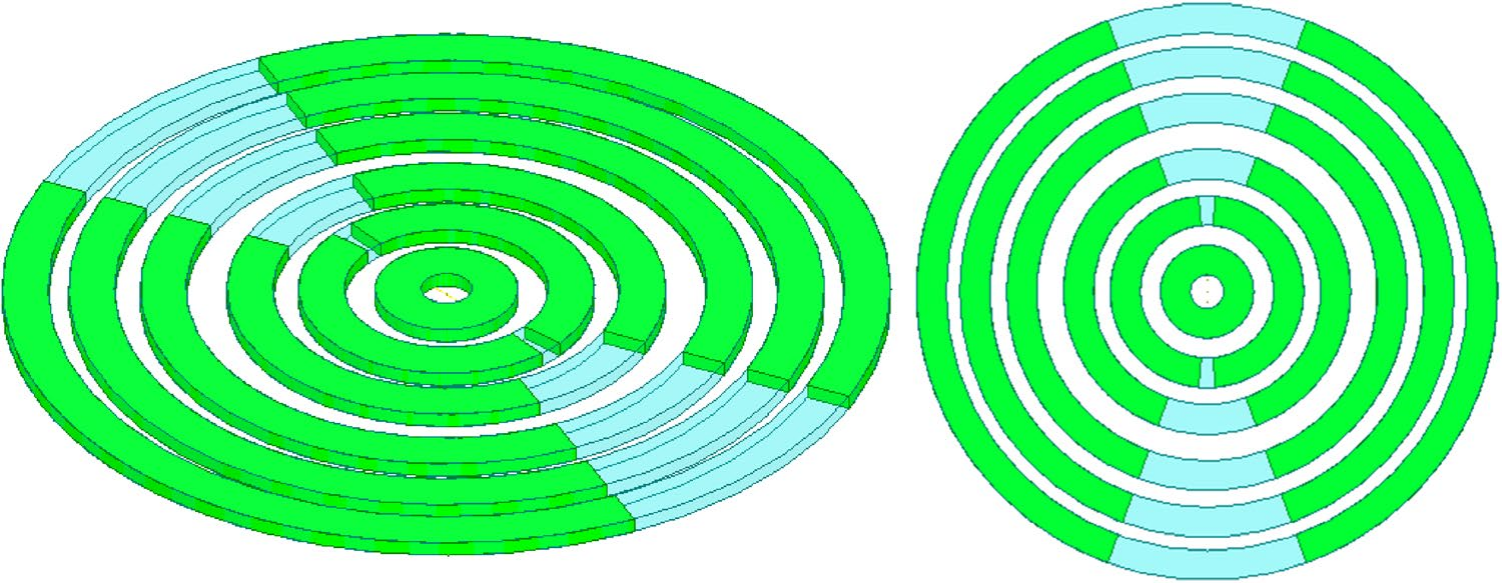
Full ring structure, isometric view (left), and top view (right).

**Figure 14:**
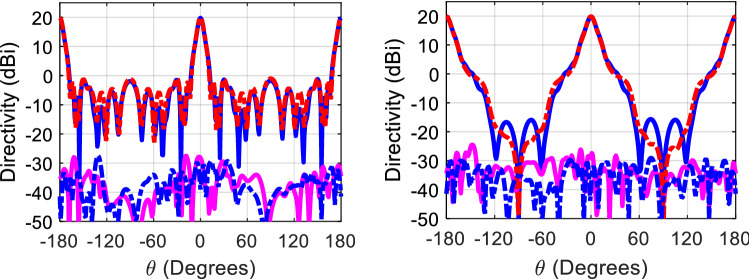
Radiation patterns for the proposed structure in Fig. 13 at 20 GHz, left (H-plane), and (right) E-plane, dotted (semi-ring), solid (full ring), blue (co-polar component), and magenta (cross-polar component).

Figure 11 shows the radiation pattern of the structure (i.e. six rings case) in principal planes when a PMC surface is used in the *xy*-plane, as can be seen back lobe radiation is suppressed and only radiation in the boresight direction exists. A directivity of 22.4 dBi is achieved in this case. Figure 12 compares the 3D radiation pattern for the case of rectangular dielectric slabs and the case of semi-ring dielectric slabs, as can be seen a 6-dB difference between the directivities exist, in addition the rectangular slab case has fan-beam like pattern while the semi-ring case has a pencil-beam like pattern as expected. At this point it is worth investigating the case of full rings rather than semi-rings, the purpose behind the use of semi-rings is that it well matches the wave-front of the infinitesimal current source. Figure 13 shows the full ring structure, the cyan region shows the complementary part of the semi-ring case. Figure 14 shows the radiation pattern for both cases (i.e. full and semi-ring cases). As can be seen the full ring performance is almost identical to the semi-ring case with no additional improvement on the directivity as expected. This suggests that the semi-ring shape is well matched to utilize the wave-front generated by the dipole.

## Circular polarization operation

**Figure 15:**
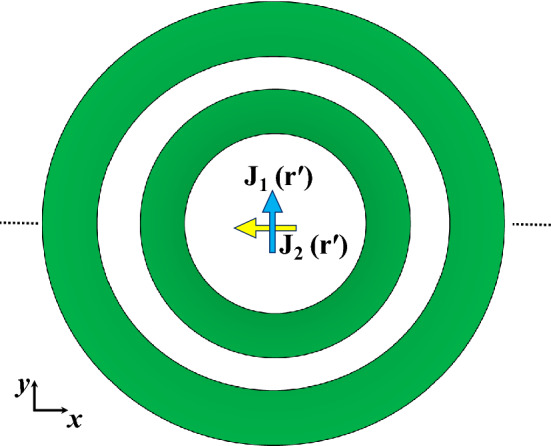
Two orthogonal current sources with 90° phase shift inside multiple dielectric rings structure.

**Figure 16:**
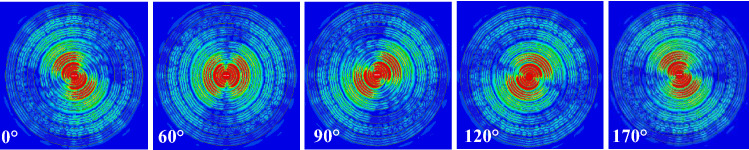
Electric field heat map for the CP source at different phases, the heat map scale is the same as in Fig. 8.

**Figure 17:**
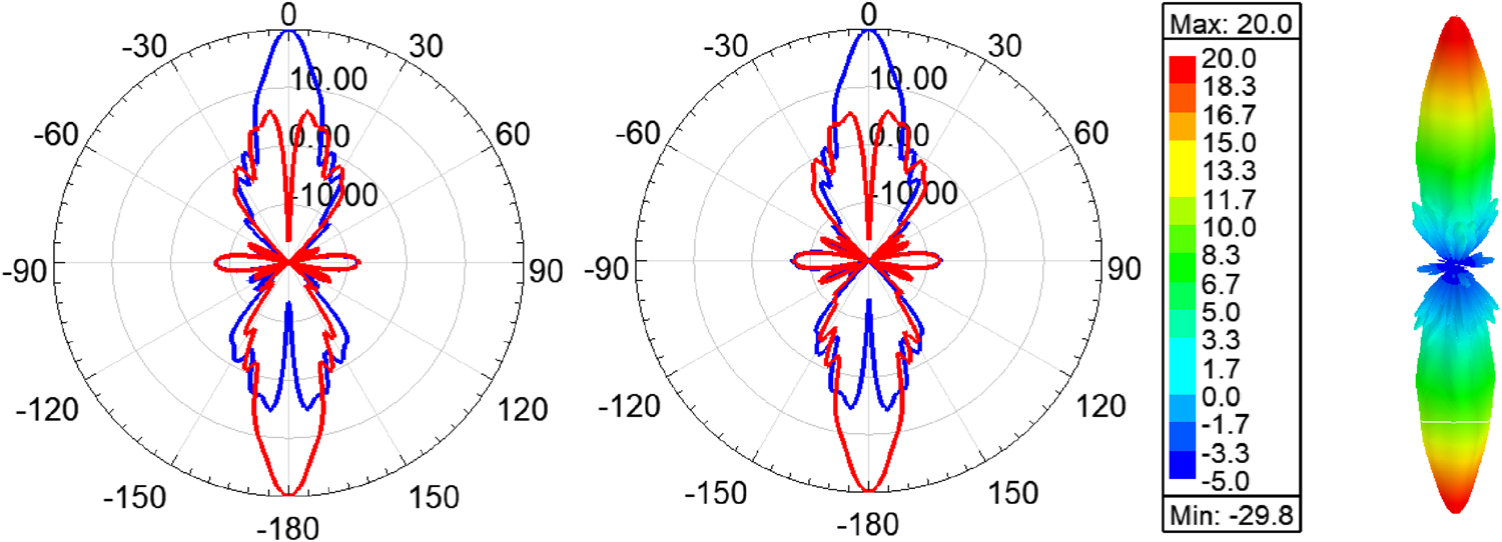
Radiation pattern at 20 GHz in *xz*-plane (left), *yz*-plane (middle), and 3D pattern (right), LHCP Co-polar (blue), and RHCP Cross-polar (red).

**Figure 18:**
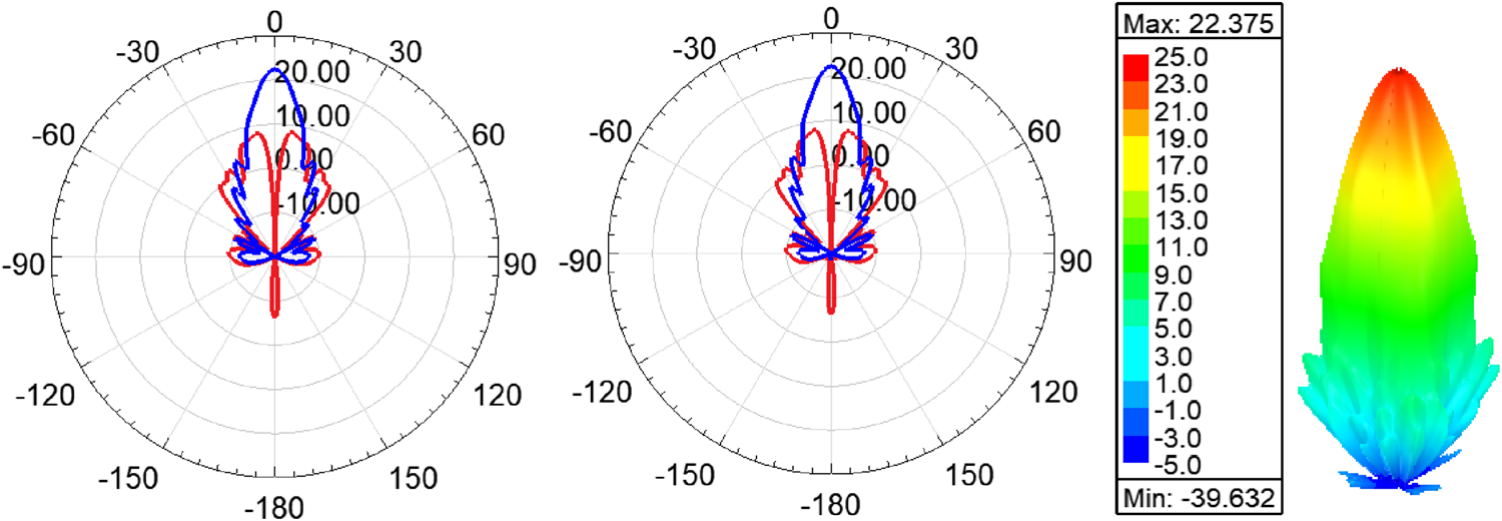
Radiation pattern in at 20 GHz *xz*-plane (left), *yz*-plane (middle), and 3D pattern (right), LHCP Co-polar (blue), and RHCP Cross-polar (red) for the PMC case.

Circular polarization (CP) is desired in several applications, a circularly polarized wave can be generated by two orthogonal current sources with 90° phase shift. This is equivalent to a rotating electric current source. This suggests that the full ring case studied previously can be utilized for circular polarization operation easily by having a CP source in the center of the dielectric rings as shown in Fig. 15. The same full ring structure in Fig. 13 was used. Figure 16 shows the electric field heat map at different phases, as can be observed the fields’ intensity rotates counter clock wise as expected. Figure 17 shows the corresponding radiation pattern. Figure 18 shows the pattern for the case backed by PMC. As can be noticed from Fig. 17, the structure radiates a Left-Handed Circularly Polarized (LHCP) wave along the positive z-direction, and a Right-Handed Circularly Polarized (RHCP) along the negative *z*-direction. Therefore the back radiation is actually the cross-polar component of the forward wave. By truncating the structure in the *xy*-plane by a PMC surface, unidirectional radiation can be obtained as shown in Fig. 18. It is also worth noting that a dual polarized source where the two current sources have different feed ports (i.e. different data signals) would have the same directive behavior as in the linear case, this can be easily observed from the symmetry around both the *x* and *y*-axis.

## Practical realization considerations

**Figure 19:**
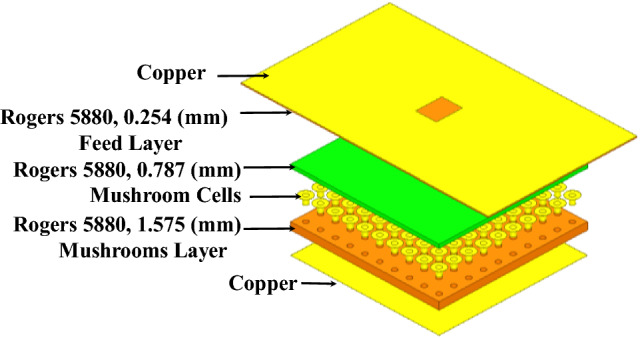
Slot excitation using printed ridge gap waveguide technology.

**Figure 20:**
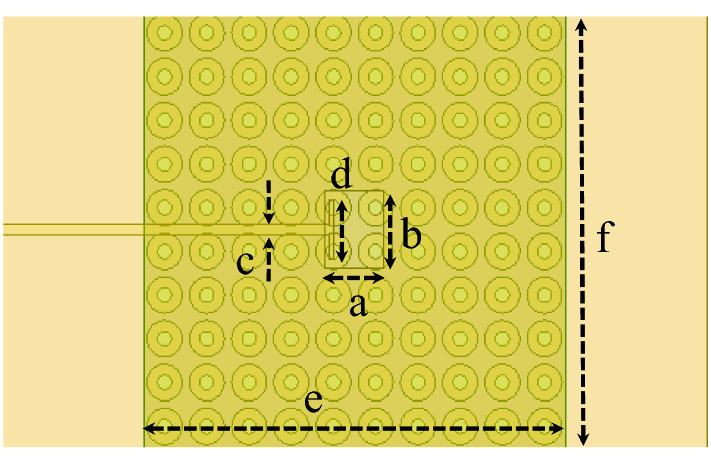
Detailed view of Fig. 19.

**Figure 21:**
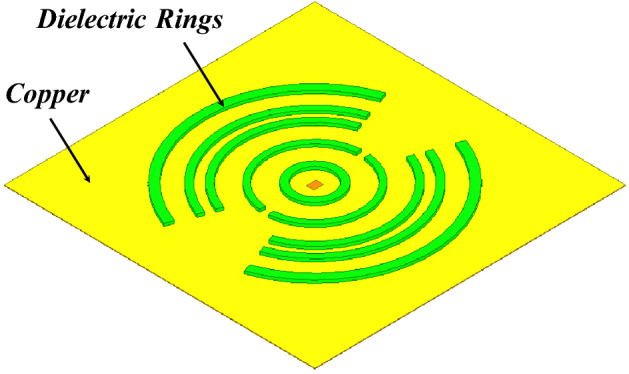
PRGW slot with semi-dielectric rings.

**Figure 22:**
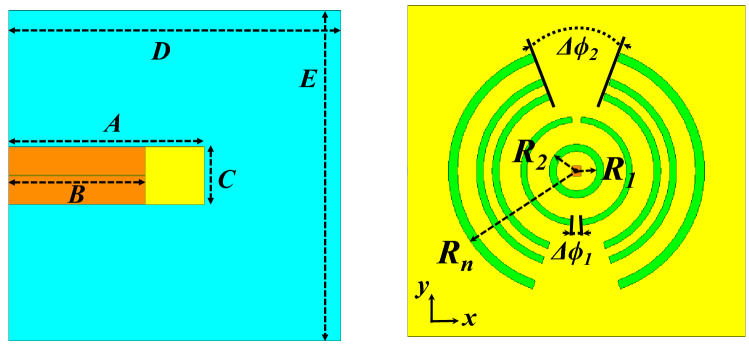
Detailed view for Fig. 21, right (top-view), and left (bottom-view).

**Figure 23:**
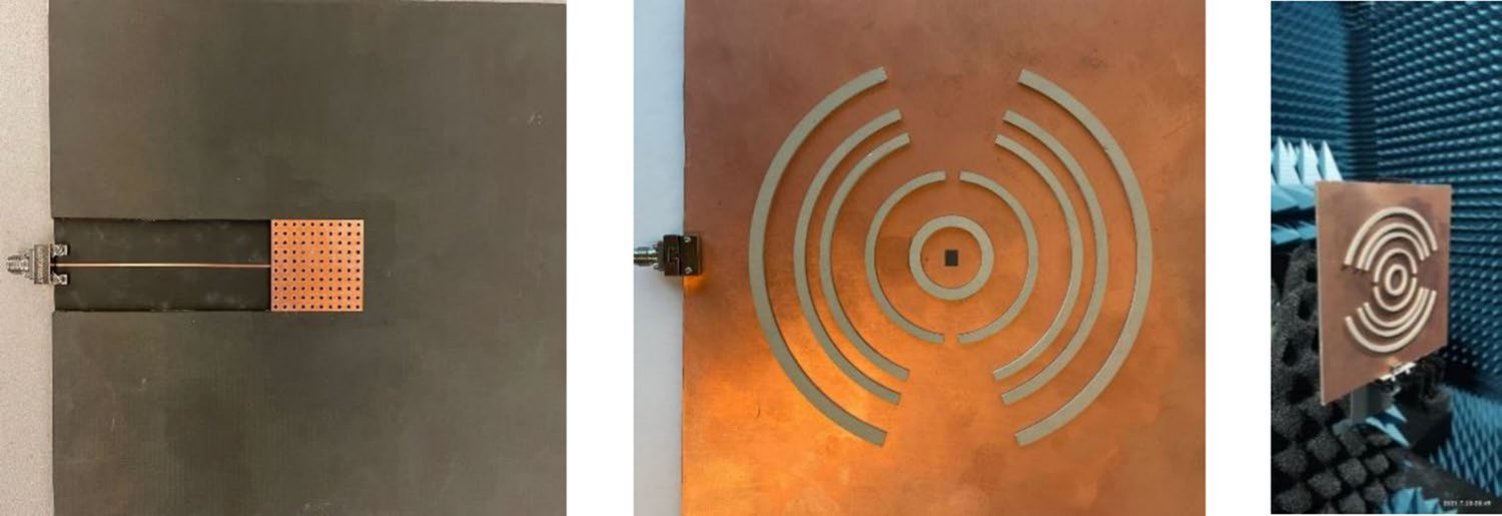
Fabricated Prototype, bottom (left), top (middle), and right (isometric view).

**Figure 24:**
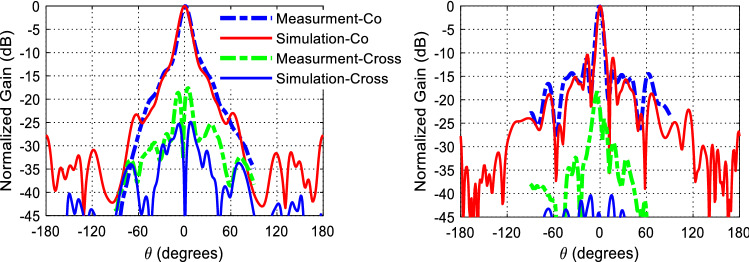
Radiation pattern of the proposed structure at 20 GHz, E-plane (right), and H-plane (left).

**Figure 25:**
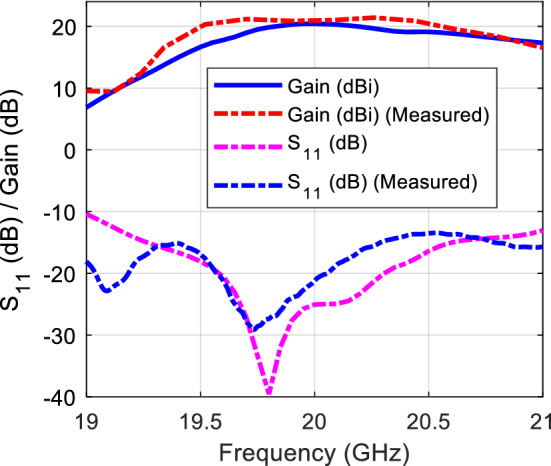
Gain and S_11_ of the proposed structure.

Dipole antennas require differential type of feeds which usually can be obtained by a Balun circuit, adding a Balun circuit usually increases the structure complexity and associated losses. Also the case presented for the dipole requires the realization of an Artificial Magnetic Conductor (AMC) using periodic structures, this also further increases the complexity of the design. Therefore, it would be easier to implement a modified version of the dual case, which uses a slot excitation with the dielectric rings. Figure 19 shows a Printed Ridge Gap Waveguide (PRGW) fed slot antenna. Figure 20 shows a detailed view. All corresponding dimensions are listed in Table 2. Such structure is fully packaged and it eliminates any parasitic radiation from the feed that might distort the radiation characteristics of the antenna. Also, as the whole structure is backed by copper, it eliminates any back radiation. The cell used is a mushroom cell, the patch diameter is 2.5 mm, and the periodicity of the cell is 3 mm. The dispersion diagram for the proposed cell is omitted for brevity. The bandgap ranges from 13 – 31.5 GHz. Many works in the literature discuss printed EBG packaging, some but not limited to are^26–29^. Figure 21 shows the PRGW slot antenna with the semi-dielectric rings, Fig. 22 shows a detailed view of the structure. The semi-rings material is made of Rogers 6010 with dielectric constant of 10.2 and a loss tangent of 0.0023. Table 3 lists the complete set of dimensions. Figure 23 shows the actual prototype for the proposed structure. Figures 24 and 25 shows the radiation patterns, gain, and S_11_. As expected a gain of 20 dBi is obtained, only 5 semi-ring pairs are used instead of 6 to maintain the structure size within the available Rogers substrate size, and to satisfy fabrication process requirements. It is worth noting that this type of antenna has a narrowband pattern stability (3% fractional bandwidth), further details can be found in^15^, nonetheless the impedance bandwidth is independent from the dielectric semi-rings, and can be set by the slot design, in this case the impedance bandwidth is 10%.

**Table 2:** Dimensions (millimeters).

a	b	c	d	e	f
4.2	5.3	0.7	4.1	30	30

**Table 3:** Dimensions (millimeters).

R_1_	R_2_	R_3_	R_4_	R_5_	R_6_	R_7_	R_8_	R_9_	R_10_
10	13.75	25	28	39	42.4	47	50.5	60	64.5
R_11_	R_12_	Δϕ_1_	Δϕ_2_	A	B	C	D	E	H_d_
74	78.8	5°	20°	100	70	30	170	170	1.5

**Figure 26:**
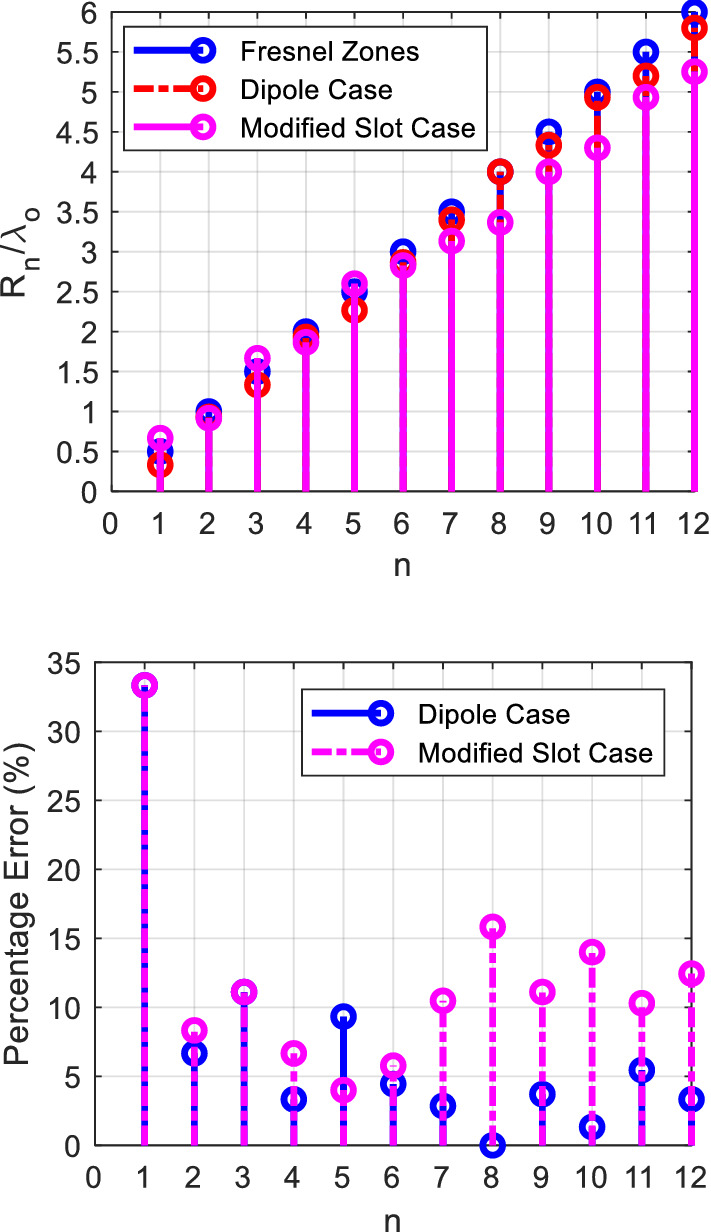
Calculated Fresnel zones compared with realized structures, and Percentage error for each case.

It is worth pointing that there is uncertainty in the gain measurement due to the use of several transitions, variations of the standard gain horn, and the visual alignment of the antennas. This creates inaccuracies and sometimes uncertainty in the range of 3 dB in the gain measurement. Figure 26 compares the calculated zones using Eq. (11), and the proposed dipole and slot structures zones. As can be seen, the first ring has the highest error (~ 35%), due to the short distance from the source, and the fact that the source is not ideally spherical, so the first ring task is to maintain the spherical behavior for the next zone, and this is achieved by the slab radiating aperture in the *yz*-plane, consequently the error in the next slabs is reduced to be less than 10% for the dipole case, and 15% for the slot case. Obviously, the slot has more deviation compared to the thin dipole. As the slot width increases to widen the impedance bandwidth, its radiation pattern deviates more from the ideal omnidirectional radiation pattern. From such analysis we can use (11) to calculate a crude value for the rings radii, and with a full-wave solver the 10–15% error can be fine-tuned to optimize the gain. Another advantage is that the dielectric material permittivity provides another degree of freedom to control the passage of the wave, where the reflectivity can be controlled by its size, and hence enable further control of the ratio of the reflected to transmitted power, this is very important for the control of power tapering and the side-lobe level.

In^15^, A detailed analysis along with a semi-analytical model using the Uniform Theory of Diffraction (UTD) was derived, the unique aspect is that UTD along with diffracted fields perspective provide a great tool to analyze and understand the behavior of such structure, further to that, this work links such understanding with the Fresnel–Huygens zones.

In general, all antennas whose aperture field distribution can be represented by one or more traveling waves in the same direction are referred to as traveling wave or non-resonant antennas. Standing wave antennas, such as dipole, can be analyzed as traveling wave antennas with waves propagating in opposite directions (forward and backward). As a matter of fact, aperture antennas, such as reflectors and horns can be treated as traveling wave antennas. Yagi-Uda is an example of a discrete element traveling wave antenna^14,30^.

A travelling wave may be classified as a slow wave if its phase velocity is equal or smaller than the velocity of light in free space. A fast wave is one whose phase velocity is greater than the speed of light. Moreover, travelling wave antennas can be classified as either, surface wave antenna “an antenna which radiates power flow from discontinuities in the structure that interrupt a bound wave on the antenna surface.” A surface wave antenna is in general a slow wave structure. Another traveling wave antenna is the leaky-wave antenna, it’s defined as “an antenna that couples power in small increments per unit length, either continuously or discretely, from traveling wave structure to free-space, Most of leaky wave antennas are fast wave structures^14,25,30–35^.

The interesting thing about this structure is that it does not require any substrate excited modes, and therefore no surface-bound waves or substrate leaky modes are involved in the radiation mechanism, simply as shown in Figs. 7 and 8, the propagating wave is a direct radiated wave from the excitation (i.e. there is no substrate), in addition to that the radiation mechanism of the structure depends mainly on the waves being propagated through the dielectric slabs, as can be seen in the heat maps in Fig. 8, the wave can propagate within the dielectric slab over multiple half-wavelengths. As the structure in Fig. 7 can be truncated by symmetry in half by PMC (Electric current source case), or PEC (Magnetic current source) surface in the *xy*-plane according to image theory, this indicates that the PMC/PEC ground plane has no effect on altering the propagating TEM wave from the source excitation (i.e. the infinitesimal electric/magnetic current source).

**Figure 27:**
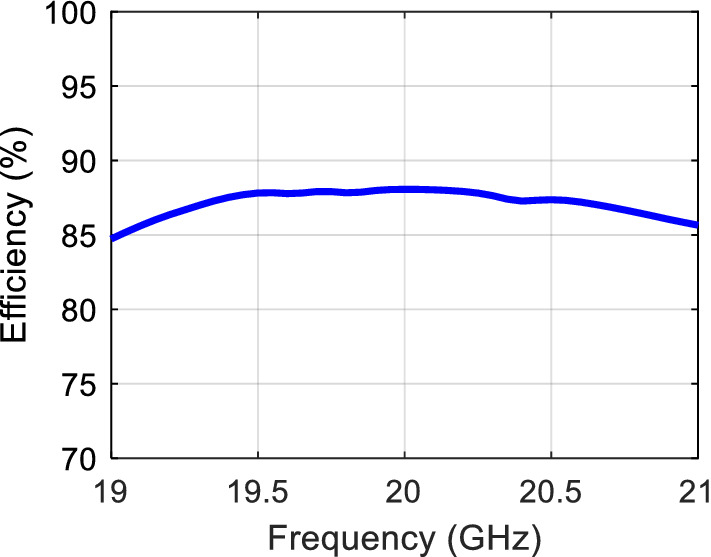
Calculated radiation efficiency.

Figure 27 shows that the calculated radiation efficiency value goes up to 88%. A 20 dBi gain is achieved with just a single feed point, and this has a positive impact on improving the radiation efficiency by avoiding the losses that would rise from the use of a power divider feeding network. Also, the solution is fully printed and doesn’t require any bulky waveguide feed or transitions, the long feed line exists only for characterization purposes, in an integrated solution, the long line can be eliminated by having the transceiver chipset in the vicinity of the antenna.

At a certain point, the structure might be seen as an antenna exciting several DRAs parasitically. Some works, as in^36–41^ studied exciting a DRA with adjacent parasitic DRAs. However, in those works, the distance between the elements is very small electrically. Where the elements couple strongly through the near field, and sometimes can be seen as a large element with air gaps in between. Those gaps can be equivalently perceived as lumped element phase shifters. In such a case, the aperture is enlarged, which enhances the gain. However, the coupling to the source alters the input impedance. On the other hand, in this work the distance from the source is increased so that it can go up to 0.4λ_o_, and the distance between the dielectrics can go up to 0.55λ_o_. This distance allows the wave detaching from the source to spread spherically in space before reaching the adjacent element. In addition, such distance from the source reduces the effect of the adjacent dielectric slabs on the input impedance of the source. This technique, therefore, can have a higher gain performance by maximizing the aperture of the antenna. In^42^, a series fed patch antenna array produced a 11.9 dBi gain with reduced side lobes; however, it required the design of a power divider feeding network, such network can be eliminated by applying the proposed concept and avoid any losses within the feeding structure.

## Conclusion and future work

A low-cost antenna solution has been proposed achieving a 20-dBi gain. A thorough analysis of the propagation mechanism accompanied by a unique physical insight has been provided. The realized structure has a low profile, low cost, and compact features. Potential future work would be studying the power tapering quantitatively to implement any distribution like Chebyshev and others.
